# Supplementation of Diets With *Spirulina* Influences Immune and Gut Function in Dogs

**DOI:** 10.3389/fnut.2021.667072

**Published:** 2021-05-28

**Authors:** Ebenezer Satyaraj, Arleigh Reynolds, Robyn Engler, Jeff Labuda, Peichuan Sun

**Affiliations:** Nestlé Purina Research, One Checkerboard Square, St. Louis, MO, United States

**Keywords:** *Spirulina*, *Spirulina (Arthrospira) platensis*, nutritional immunology, immunity, gut health and function, dog

## Abstract

*Spirulina* refers to two species of blue green algae (*Arthrospira platensis*, and *A. maxima*) consumed by humans as food for centuries. While, *Spirulina* has been shown to have immune enhancing properties in several animal and human studies, there are no systematic studies in dogs. The aim of this study was to evaluate the immunomodulatory effect of dietary supplementation with *Spirulina* in dogs. The study was conducted in two phases: Pre-test (8 wks.) and Test (42 wks.). Thirty adult dogs (mean 2.9 yrs.) were randomized into two groups and fed a nutritionally complete diet in the “Pre-test” phase. At the end of “Pre-test” phase all dogs received a rabies vaccine, and dogs in “test group” were switched to diet supplemented with dried *Arthrospira platensis* (*Spirulina*). Response to rabies vaccine was evaluated by Rapid Fluorescent Focus Inhibition Test (RFFIT). Gut immune response was assessed by measuring fecal IgA. Gut microbiota was evaluated by Temporal Temperature Gel Electrophoresis (TTGE) methodology. Repeated measures ANOVA was used to test for differences between groups and statistical significance considered to be *p* < 0.05. Dogs fed diets supplemented with *Spirulina* demonstrated enhanced immune status by showing significantly higher vaccine response and higher levels of fecal IgA as compared to the control group. Supplementing diets with *Spirulina* also resulted in significantly increased gut microbiota stability in the test group. In conclusion, diets supplemented with *Spirulina* significantly enhanced immune response and gut health in dogs.

## Introduction

*Spirulina* are planktonic blue green algae that grows naturally in warm alkaline lakes in subtropical and tropical areas of America, Mexico, Asia and Central Africa. It has been the traditional diet of some in Africa and Mexico for many centuries (1). There are 3 main *Spirulina* algae—*Spirulina platensis, Spirulina maxima*, and *Spirulina fusiformis*- that have been most regularly investigated for their nutrition and functional properties (2, 3). Although these algae were firstly identified as belonging to the genus *Spirulina*, they are now accepted to be of the genus *Arthrospira*, however the name *Spirulina* has remained for historical reasons. *Spirulina* has been recognized as having a unique nutritional profile with a very high protein content (60–70% of dry matter content), and being rich in vitamins, minerals, essential fatty acids particularly gamma-linolenic acid, as well as other bioactive components (4). This rich nutritional profile has earned *Spirulina* an endorsement from both the National Aeronautics and Space Administration (NASA) and the European Space Agency (ESA) as a food suitable for long space missions. Over the last number of years, studies have identified many positive benefits of *Spirulina*, including immunostimulatory, antioxidant, anti-inflammatory, anti-viral, and anti-bacterial effects (4–6), to name some of the most important. We have published widely in the area of nutritional regulation of immune and gut health in dogs and cats and hypothesized that inclusion of *Spirulina* to companion animal pet foods could offer many health promoting benefits.

Studies have shown that *Spirulina* can modulate both cellular and humoral immune responses. In terms of cellular immune responses, there are several reports of *Spirulina* having a specific action on monocytes and natural killer (NK) cells, components of the innate immune system. In chickens and humans, macrophage phagocytic response was enhanced, and NK cell activity increased, in response to *Spirulina* administration (7–9). The phagocytic activity of macrophages isolated from cats was also found to be increased in response to antigen exposure in the presence of *Spirulina* (10). In dogs and mice, a polysaccharide extract of *Spirulina platensis* could increase white blood cell numbers when the haematopoietic system was damaged by irradiation (11). Mechanistically spray dried *Spirulina platensis*, orally administered to healthy male volunteers, increases IFN-γ production and phagocytic activity of isolated NK cells stimulated with IL-12/18. In the above study, *Spirulina* enhanced Toll like receptor (TLR)-2 and 4 mediated production of IL-12 from peripheral blood mononuclear cells, thus indicating *Spirulina* first activates monocytes and macrophages to produce cytokines that stimulate NK cells (9). An action through TLR-2 but not -4, leading to NF-κB activation, was further suggested in studies in human monocytes (6), while a very recent study has again implicated TLR-4 (12).

*Spirulina* also modulates humoral immune responses. Treatment of mice with *Spirulina* for 4 weeks enhanced *ex vivo* production of IgA from Peyer's patch cells on antigen presentation (13). A polysaccharide extract of *Spirulina* similarly increased mouse Peyer patch IgA production (6). The authors of this polysaccharide study suggested that the IgA stimulatory effect may have occurred via an increase in numbers of CD11b(+) dendritic cells or via increased IL-6 production (6). Broiler chickens fed a *Spirulina* supplemented diet demonstrated a higher antibody-specific response against injected sheep red blood cells (SRBC) (injected as an antigen) (7). Antibody production is critical in allergic reactions. *Spirulina platensis* phycocyanin extract may protect against allergy by suppressing antigen specific IgE and IgG responses and upregulating mucosal IgA response, while suppressing antigen induced small intestine inflammation (14). The above studies clearly indicated that *Spirulina* has strong potential to improve intestinal humoral immunity and thereby protecting against infection and potentially allergy.

In addition to its immunostimulatory effects, *Spirulina* is rich in β-carotene and tocopherols, nutrients of proven antioxidant and anti-inflammatory properties. *Spirulina* has been shown to reduce oxidation in brain, plasma and liver extracts (15). Phycoyanin extracted from *Spirulina platensis* can act as a free radical scavenger, iron chelator and protects the activity of anti-oxidant enzymes (5, 16). Further *in vitro* studies demonstrated *Spirulina* could alleviate oxidative damage associated with the cancer drug Flurouracil (17), as evidence by a reduction in oxidative production of malondialdehyde. Oxidation and inflammation play a key role in many diseases including degenerative diseases. *In vivo, Spirulina* can reduce markers of brain oxidative damage and reverse age-related increases in proinflammatory cytokines (18). While in a mouse model of Parkinson's disease, a *Spirulina* enriched diet was found to be neuroprotective (19). These antioxidant and anti-inflammatory effects seem to translate to clinical settings as studies in humans found the oral *Spirulina* administration helped to elevate symptoms of allergic rhinitis such as nasal congestion and itching (20, 21).

Given its immune enhancing, antioxidant and anti-inflammatory effects, it is not surprising that there is emerging evidence that *Spirulina* can modulate the gastrointestinal microbiota (22). We have previously investigated modulations of the gut microbiota as a result of dietary supplementation with bovine colostrum extract in both dogs (23) and cats (manuscript under review), and as a result of probiotic supplementation with *Enterococcus faecium* SF68 in dogs (24). Our investigations clearly demonstrated that dietary modulations are a highly effective route to not only stimulate both intestinal and systemic immune function, but also improve intestinal microbiota stability. In this current study, we evaluated the immune and gut health benefits of supplementing diets with *Spirulina* in dogs. To our knowledge a study of this type with *Spirulina* has never been carried out in dogs.

## Materials and Methods

### Animals and Diets

**Figure 1:**
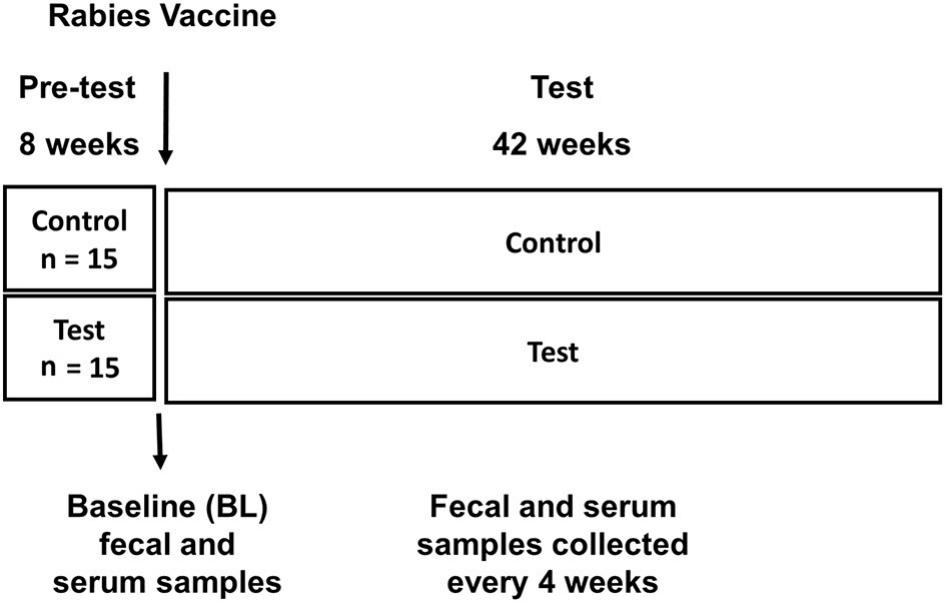
Experimental design: Pre-test phase and Test phase are depicted, along with timing of rabies vaccination, baseline (BL) sample collection, sample collection during test phase.

Thirty adult dogs (Husky crosses, 2–7 years, mean 2.9 years) were used in this study. During an 8-week pre-test period all dogs were fed a commercially available nutritional complete and balanced dog food [Nestlé Purina product: ~29% protein, 36% carbohydrate, 19% fat; 1.4% fiber, 3894 ME Kcal/Kg)] (Supplementary Table 1). At week 0, dogs were vaccinated with a rabies vaccine (IMRAB® 3, Merial Inc, Duluth, GA) as part of their normal veterinary care. At this time point dogs were randomly assigned into two feeding groups (*n* = 15 per group) based on sex, age, and fecal IgA (used as a marker of immune status). Dogs in the control group continued to be fed the “Control” pre-test diet and the other half fed the “Test diet” which was pre-test diet supplemented with 0.2% spray-dried *Spirulina* (*Arthrospira platensis*, Cyanotech Corporation, Kailua-Kona, Hawaii, USA) (Figure 1). Dogs were fed twice per day, once in the morning and once in the evening. Food intake was recorded daily, and body weight was recorded weekly. All dogs were fed their respective diet until the end of the study.

Dogs were exercised 3 days a week during the 42 week study, using a routine that was been previously described (23). The trial protocol was conducted in strict accordance with the guidelines established by the Nestlé Purina Pet Care (NPPC) Advisory Committee. Jugular or femoral blood samples were collected every 4 weeks from week 0 to week 42 (using BD Vacutainers with sodium citrate as the anticoagulant, Becton & Dickenson) (Figure 1). To obtain plasma, blood samples were centrifuged at 10,000 rpm for 10 min at 6°C and plasma stored at −80°C until assayed for immune markers. Fecal samples were collected every 4 weeks, processed and immediately stored in a −80^0^C freezer (Figure 1). Fecal score was recorded daily and graded using a seven-point scale with a score of 1 representing firm, hard feces and a score of 7 representing liquid diarrhea (Supplementary Table 2). A score of 2 or 3 is considered ideal.

### Measurements of Antibodies in Plasma

A Rapid Fluorescent Focus Inhibition Test (RFFIT) was used to measure serum rabies virus neutralizing antibodies. This is a functional assay and measures the ability of antibodies in the serum to neutralize rabies virus, and hence is a good reflection of how effectively the animal would be able to ward off a potential infection with the rabies virus. The test was carried out by the Rabies Laboratory of Kansas State University according to a method previously published (25).

### Measurements of Antibodies in Feces

Secretary IgA (sIgA) levels in fecal samples were measured as an indicator of gut-associated lymphoid tissue (GALT) activity using an ELISA method as previously described (23). Briefly, protein was extracted from fecal samples using extraction buffer and the supernatants were collected and frozen at 80°C until assayed for IgA by 96 well ELISA using mouse anti-canine IgA (Serotec, Raleigh, NC) and a secondary antibody of polyclonal goat anti-canine IgA conjugated with HRP (Serotec, Raleigh, NC). Color development was done with 3,3′,5,5′-Tetramethylbenzidine (TMB) peroxidase substrate (KPL, 50-76-00) according to the manufacturer's instructions. Color development was read at 450 nm and results were expressed as μg/ml using a feline IgA standard. Values for fecal IgA were normalized with the total protein content. The total protein content was measured using a BCA Protein Assay Kit (Pierce, 23225).

### Measurement of C-Reactive Protein

C-reactive protein (CRP) is an acute phase protein produced by the liver in response to inflammation. It is recognized as a sensitive marker of tissue damage and inflammation_._ CRP was measured as a general marker of inflammation to confirm that any immune enhancement was not a result of, or did not lead to, a generalized inflammation or non-specific immunostimulation. Serum CRP level was measured in all dogs toward the end of the trial using a canine CRP kit (BD Canine CRP ELISA Kit; BD Bioscience), according to the manufacturer's instructions.

### Measurement of the Effect of *Spirulina* on Gut Microbiota

At week 42, after 2 days of rest, all dogs participated in their standard exercise program. Dogs ran 10 miles in a harness pulling an unladen sled. Each team contain approximately equal numbers of control and *Spirulina* supplemented dogs. There was no difference in time to complete the task between teams (average 33 min and 30 s). This exercise was used as an inducer of physiological challenge to examine the effect of *Spirulina* on the gut microbiota stability. Rectal swabs were taken 24 h before (“pre”-samples) then 24 h after (“post”-samples) the exercise protocol. Samples were immediately snap frozen and later microbiota modulations were then assessed by Temporal Temperature Gel Electrophoresis (TTGE) (23). TTGE analysis allows the separation of 16S rRNA gene fragments that have been amplified by PCR and is a commonly used technique to identify microbiota microbial profiles (26) (described below). The “pre” samples collected prior to exercise were used to characterize the dogs baseline species diversity and evenness, as previously described (23). The effect of exercise in each of the dietary groups was assessed by comparing the per cent similarity of the “pre”– and “post”-exercise TTGE profiles. Similarity scores of the *Spirulina* supplemented group where compared to the control group (Supplementary Table 3).

### Temporal Temperature Gel Electrophoresis

TTGE analysis was used to identify modulations in microbial profile of fecal samples in association with dietary supplementation with *Spirulina* as time points as described above. TTGE analysis was performed on extracted DNA according to the method that has been previously published (23). Gel images were captured and digitized using the FMBIOII (version 1.1) software (Hitachi). Digitized images were analyzed using the GelCompar II (version 2.0) gel analysis software (Applied Math). Band classes were established and band densities (based on height and band surface) within each class were tabulated. Each band class contained all the bands that migrated to the same adjusted location on the gels.

### Statistical Analysis

Data are presented as mean ± SEM. Repeated measures ANOVA was carried out using Statistical Analysis Systems statistical software package SAS (SAS Institute, Cary, NC, USA), to test overall differences between groups for all measures. Dunnett's test was used to adjust for multiple comparisons with the control group. For all tests, significance was considered at *P* < 0.05.

## Results

### General Physiological Status

At the start of the trial the average weight of the dogs in the test group was 21.6 ± 0.6 kg and in the control group 22.05 ± 0.7 kg. Food intake and body weight did not differ between the two groups during the trial (data not shown). There was no significant difference between control and the *Spirulina* supplemented diet on all blood chemistry parameters measured (data not shown). No significant difference in fecal scores was also observed between dietary groups (data not shown). Levels of CRP, a marker of inflammation, measured toward the end of trial, were well within the normal range (0.8–16.4 μg/ml) (27) [0.04 ± 0.01 μg/ml for control, and 0.03 ± 0.01 μg/ml in the *Spirulina* supplemented group at end of trial].

### Immune Response in the Gut

**Figure 2:**
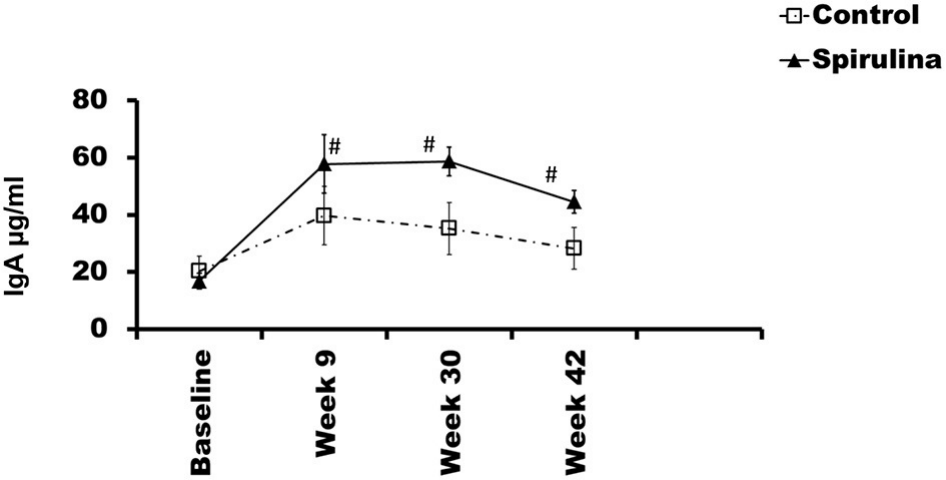
GALT response: Changes in fecal IgA in each group during the trial are depicted as mean fecal IgA values ± SEM. X-axis depicts time in weeks and Y-axis depicts mean fecal IgA values in μg/ml. ^#^*p* < 0.05.

Fecal secretory IgA levels were analyzed by ELISA. Repeated measures ANOVA demonstrated a significant effect of diet on levels of sIgA (*P* < 0.05) (Figure 2). Fecal IgA levels were significantly increased at weeks 9, 30, and 42 in the *Spirulina* fed group compared to the control group (*P* < 0.05) (Figure 2).

### Response to Rabies Virus Vaccine

**Figure 3:**
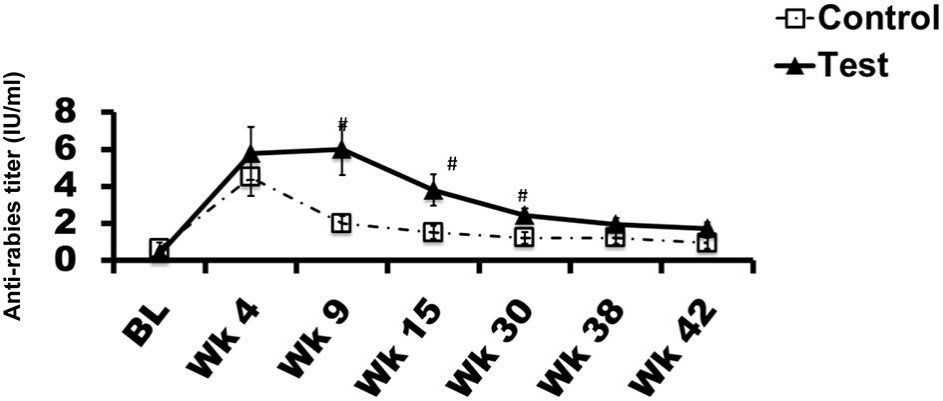
Rabies virus vaccine response: Antibody level ± SEM in each group during the trial is plotted. X-axis represents time in weeks and Y-axis represents anti-rabies virus titers in IU/ml. ^#^*p* < 0.05. BL denotes baseline value.

All dogs received a rabies vaccination at week 0. The vaccine is commercially available, approved for use in dogs, routinely used in veterinary practice, and was chosen in consultation with the veterinarian. Repeated measures ANOVA demonstrated a significant effect of diet on antibody response (*P* < 0.05). In comparison to the control diet, the *Spirulina* supplemented group had significantly higher antibody levels at weeks 9, 15, and 30 (Figure 3, *P* < 0.05).

### Effects of *Spirulina* on Gut Microbiota

Using TTGE microbial profiling, dogs' gut microbiota patterns were compared before and after exercise. Exercising induces a certain amount of physiological stress on the dogs. When the “pre”-exercise microbiota pattern (collected 24 h before exercise) was compared with the “post”-exercise microbiota pattern, dogs fed the test diet supplemented with *Spirulina* had increased gut microbiota stability as evidence by increased similarity in the “pre” and “post” banding pattern 77.8 ± 2.3 vs. 48.6 ± 3.0% compared to the control group, *P* < *0.05*).

## Discussion

In this study we have demonstrated that 0.2% *Spirulina platensis* powder supplemented in the diet of dogs was associated with significantly enhanced immune and gut health. *Spirulina* increased fecal IgA levels after just 9 weeks of feeding, and this effect was maintained throughout the study. Gut microbiota maintained enhanced stability following a challenging exercise in the *Spirulina* fed dogs as compared to the control group. Systemic immune responsiveness was found to be fortified with *Spirulina* feeding leading to a faster and stronger induction of rabies vaccine titer. Together these results suggest that *Spirulina* fed dogs have a healthier and more robust immune system.

We previously demonstrated that increased fecal sIgA levels could be induced with the dietary inclusion of a immunomodulating agents such as bovine colostrum (23) or the lactic acid probiotic *Enterococcus faecium SF68* (24). We now extend this finding to *Spirulina platensis* and provide further evidence of the health benefits of immunonutrition for companion animals. IgA is the most abundant immunoglobulin produced by the GALT, and increased fecal sIgA content is indicative of increased GALT activity (28). IgA plays an important role in defending against pathogenic infection, in preventing antigens from entering the epithelium, and in the selection and maintenance of colonizing bacteria (29). Intestinal IgA's primary role is to maintain intestinal homeostasis and to protect mucosal membranes from infection by pathogenic microorganisms and enteric toxins by inducing a process known as immune exclusion (30). IgA maintains immune exclusion through a number of ways, for example, by binding to antigens trapping them in the mucus and preventing their binding to cell surface receptors, and by reducing bacterial virulence (31). In dogs, a reduction in fecal sIgA has been shown to be associated with some chronic enteropathies (32, 33), suggesting fecal sIgA level is an indicator of intestinal immune health. At a mechanistic level, it is known that *Spirulina* can have a direct action on intestinal immune cell function and one mechanism explored includes the activation of intestinally located TLR's (6, 9). A previous study has demonstrated that constitutive activity of TLR-4 in the intestinal epithelium of mice lead to B cell recruitment, their switching to IgA+ cells, and consequently increased fecal IgA levels (34).

Previous studies have also shown that *Spirulina* or extracts of *Spirulina* could enhance gut IgA levels (13, 14). In mice orally administered shrimp extract as an antigen, intestinal IgA levels were shown to be increased in *Spirulina* as compared to control fed mice (13). In another study, ovalbumin was administered to mice to induce antigen-specific antibodies in their lymphoid tissues. This induced both antigen specific IgA and total IgA in the Peyer's patch however, the effect was significantly enhanced in an ovalbumin and phycocyanin combined group compared to ovalbumin alone. In the serum, phycocyanin prevented the antigen induced increase in IgG1 and IgE levels, and suppressed ovalbumin induced inflammation of the intestine (14). The authors of this mouse study proposed that phycocyanin, extracted from *Spirulina platanis*, may reduce allergic inflammation by supressing antigen specific IgE and augmenting intestinal IgA production. The production of antigen specific sIgA from IgA-secreting cells in Peyer's patches, and accompanying higher fecal sIgA levels, has been suggested to be a mechanism for the development of food tolerance (35). Therefore, by supporting intestinal immune function, *Spirulina*, may not only help reduce opportunistic infections but may also help prevent food intolerance. In our current study, fecal sIgA levels were increased after just 2 months of feeding *Spirulina* and the positive enhancement was maintained to the end of the 42-week study. Interestingly in a human study of oral *Spirulina* administration, immunostimulatory effects of *Spirulina* were evident up to 4 weeks after the end of administration (9), indicating the dogs included in this study could have continued gut health benefits even after the end of the trial. In this study, Hirahashi et al., demonstrated that spirulina likely acts monocytes to induce IL-12, which in turn drives NK cells to produce IFN gamma and activate downstream T cell function.

The health of the immune system can be evaluated by how subjects respond to exercise (23), as exercise can temporally lower immune status (36). The intestinal microbiota is dynamic and temporal variations of content occur over time in response to life events such as stress, age, illness, and even exercise (37). We have previously shown that a more stable gut microbiota can resist exercise induced changes in gut microbiota (23). Like our previous study, we were also interested to determine if *Spirulina* could improve gut microbiota stability. At 42 weeks, all dogs participated in an exercise protocol involving a 2-day rest period followed by a 10-mile sled run. The *Spirulina* fed group had greater gut microbiota stability following exercise compared to the control group (*P* < 0.05), as measured by a greater degree of microbiota similarity before and after exercise. Although we did not determine which specific species of gut microbes were modulated by *Spirulina*, a previous *in vitro* study has shown that *Spirulina* can increase the growth of lactic acid bacteria (38). Lactic acid bacteria are normal residents of canine gut microbiota (39) and lactic acid probiotics have a safe and effective history in dogs (24, 40). By contrast, gut microbiota species diversity was not modulated by *Spirulina* supplementation, which is in line with the only other previous study that examined the effect of *Spirulina platensis* on the gut microbiota and were mice similarly showed no change in gut microbiota diversity (22). Interestingly, *Spirulina* has also previously been shown to prevent oxidative damage to skeletal muscle normally associated with exercise in untrained subjects (41), which together with this current finding indicates *Spirulina* is of significant benefit to negate the negative impacts of high endurance activity.

It is currently unclear through which mechanism *Spirulina* would influence the gut microbiota, however, the overall improvement in gut microbiota stability is likely to be connected its improvement gut immune status. The gut microbiota and gut immune system are critically interlinked. For example, the intestinal microbiota increases the proliferation of IgA synthesizing plasma cells in the intestine (42), while mucosal IgA enhances the homeostasis of gut commensal microbiota (43). Thus, beneficial effects of *Spirulina* on gut immunity will reciprocate on the gut microbiota, and vice versa. Marine algae are known to produce soluble polysaccharides which escape conventional digestion and are fermented by the gut microbiota (44). Extracts of *Spirulina platensis* have been shown to stimulate lactic acid bacteria growth (38) and can increase probiotic yields in a dairy products (45), thus indicating soluble polysaccharides of *Spirulina* may similarly escape conventional digestion, but rather reach the colon and provide substrates for microbiota growth.

Here we vaccinated the dogs with a rabies vaccine just prior to them starting the dietary trial with *Spirulina*. Dogs in both the control and *Spirulina* dietary groups mounted an immune response to the vaccination as evidenced by the production of rabies vaccine specific antibodies. However, the response antibody response was faster and stronger in the *Spirulina* group (*P* < 0.05). Similarly, one previous study in mice demonstrated an increased tetanus toxoid vaccine response 21 days post vaccination, in mice orally administered with *Spirulina* powder for 7 days prior to, and thereafter, vaccination compared to control mice who did not receive *Spirulina* (46). We have previously used co-incident vaccination protocols with dietary manipulations to understand how a given diet could influence systemic immunity as vaccine responses are a relevant biomarkers of an immunological response to challenge (23, 24), and can be interpreted as a surrogate marker of a typical immune response to infection (47). A poor response to vaccination can lead to higher rates of clinical illness (48, 49). Here *Spirulina* supplemented dogs are likely to have a better resilience to infection through the strengthening of their immune system. However, the normal CRP levels found in both control and *Spirulina* supplemented dogs clearly demonstrates that there was no overt pro-inflammatory response occurring, indicating the enhancement of immune functioning occurs in a targeted and challenge specific manner.

In conclusion, this study demonstrates for the first time that *Spirulina* can regulate the mucosal and systemic immune response of dogs. Gut microbiota stability is also regulated by its supplementation. This study further supports the use of functional ingredients in the diets of companion animals.

## Data Availability Statement

The original contributions presented in the study are included in the article/Supplementary Material, further inquiries can be directed to the corresponding author/s.

## Ethics Statement

The animal study was reviewed and approved by Nestlé Purina Pet Care (NPPC) Advisory Committee.

## Author Contributions

ES and AR designed the study, interpreted the results, and prepared the manuscript. RE, PS, and JL carried out the assays described in the study. ES and AR had primary responsibility for final content. All authors read and approved the final manuscript.

## Conflict of Interest

All authors are employed by Nestle Purina Research which funded the study in full.

## Abbreviations

GALT: gut-associated lymphoid tissue
TTGE: temporal temperature gel electrophoresis.
