# Supplementary material for: Supplementation of Diets With Spirulina Influences Immune and Gut Function in Dogs
Source: Front Nutr. 2021 May 28;8:667072. doi: 10.3389/fnut.2021.667072 (PMC8192834; doi:10.3389/fnut.2021.667072)
Supplement: Supplementary file 1 [file Data_Sheet_1.pdf]

Supplementary Tables

Table 1: **Control Diet Composition**

**Proximates**	%
Protein	29
Carbohydrates	36.1
Fat	19
mouisture	8
Ash	6.5
Crude Fiber	1.4
Total	100

Table 2 **Fecal Quality**: Fecal quality was recorded daily during the trial using a seven-point scale with a Score of 1 representing firm, hard feces and a Score of 7 representing liquid diarrhea. On this scale scores of 2 or 3 are ideal.

**Fecal Quality Scoring System**	
**Score**	**Description of Feces**
1	Very hard and dry; requires much effort to expel from body; no residue left on ground when picked up. Often expelled as individual pellets.
2	Firm, but not hard; should be pliable; segmented appearance; little or no residue left on ground when picked up.
3	Log-like; little or no segmentation visible; moist surface; leaves residue but holds form when picked up.
4	Very moist (soggy); distinct log shape visible; leaves residue and loses form when picked up.
5	Very moist but has distinct shape; present in piles rather than as distinct logs; leaves residue and loses form when picked up.
6	Has texture, but no defined shape; occurs as piles or as spots; leaves residue when picked up.
7	Watery, no texture, flat; occurs as puddles.

Table 3: **Similarity Scores:** TTGE microbial profile similarity scores of gut microflora before and after exercise in the *Spirulina* supplemented group and the control group.

***Spirulina* Supplemented Group**		**Control Group**	
**Dog ID**	**Pre vs. 24 hrs Post**	**Dog ID**	**Pre vs. 24 hrs Post**
Kona	70	Fire	50.7
Maui	85	Austin	47.3
Sara	83.2	Savannah	54.2
Dover	87	Aggie	47.32
Banshee	82	Hollywood	45
Flo	83.4	Phoenix	47
Elan	73.4	Nullah	37.8
Salomon	80.8	Dyna	50.7
Atomic	77.3	Jones	33.2
Bo	79.9	Deigo	54.6
Don	80.4	Sparrow	47.4
Tia	55.31	Rondy	40.1
Tozier	82.3	Cordova	46.9
Scarlett	83.7	Muldoon	85
Lena	63.24	Laz	41.2
